# Retaining Skin Barrier Function Properties of the Stratum Corneum with Components of the Natural Moisturizing Factor—A Randomized, Placebo-Controlled Double-Blind In Vivo Study

**DOI:** 10.3390/molecules26061649

**Published:** 2021-03-16

**Authors:** Johannes Schleusener, Andrew Salazar, Jörg von Hagen, Jürgen Lademann, Maxim E. Darvin

**Affiliations:** 1 Center of Experimental and Applied Cutaneous Physiology, Department of Dermatology, Venerology and Allergology, Charité—Universitätsmedizin Berlin, Corporate Member of Freie Universität Berlin and Humboldt-Universität zu Berlin, Charitéplatz 1, 10117 Berlin, Germany; johannes.schleusener@charite.de (J.S.); juergen.lademann@charite.de (J.L.); 2 Merck KGaA, Frankfurterstr. 250, 64293 Darmstadt, Germany; andrew.salazar@merckgroup.com (A.S.); joerg.von.hagen@merckgroup.com (J.v.H.)

**Keywords:** confocal Raman microspectroscopy, skin hydration, water binding, lipid organization, lamellae

## Abstract

The influence of a topically applied formulation containing components of natural moisturizing factor (NMF) on barrier-related parameters of the stratum corneum (SC) was investigated in vivo using confocal Raman microspectroscopy in a randomized, placebo-controlled double-blind study on 12 volunteers for 14 days. This method allowed for the elucidation of subtle differences between the verum and the placebo even though the components of the verum naturally occur in the SC. This differentiation is not possible non-invasively by conventional methods. In this study, we found that the applied verum and placebo formulations disrupted the equilibrium of water, NMF and lipids in the SC. The adverse effects of the formulation could be mitigated by incorporating it into a simplified supplementation of NMF molecules. As a long-term effect, the amount of strongly bound water increases at 30–40% SC depth (*p* < 0.05) and the amount of weakly bound water decreases at 30–40% SC depth (*p* < 0.05) for the verum. This supplement was also unexpectedly able to prevent intercellular lipids (ICL) disorganization in selected depths. In the long term, the verum treatment limited the lateral disorganization of the ICL to the upper 20% SC depth. Further research is required to elucidate the interplay of these factors in the SC, to better understand their contribution to the equilibrium and barrier function of the skin. This understanding of the interaction of these naturally occurring components could help in the future to develop and optimize topical treatments for diseases like psoriasis, atopic dermatitis, ichthyosis where the skin barrier is disrupted.

## 1. Introduction

The skin barrier function plays an important role in the regulation of the cutaneous hydration status and controls the penetration of xenobiotics [1,2,3,4]. It is mostly manifested in the stratum corneum (SC), the outermost ≈10–20 µm thick layer [5] of the skin, which is composed of flattened and denucleated cornified keratinocytes (corneocytes) embedded in a matrix of intercellular lipids (ICL), organized in bilayers/lamellae [6,7]. ICL, which consists of cholesterol, free fatty acids, ceramides and a small fraction of cholesterol sulfate [8], can be characterized in lateral and lamellar organization, which is non-homogeneous throughout the SC [9,10,11]. A higher ordering (orthorhombic) entails a higher skin barrier function, while a lower ordering (hexagonal) describes a lower skin barrier function [12]. The lipid chain length, which can be ≈6 nm (short periodicity phase) or ≈13 nm (long periodicity phase), was also found to be related to skin barrier alterations [9]. Most methods to determine the lipid ordering in the SC, such as (cryo-) electron diffraction [13], differential scanning calorimetry [14,15], X-ray diffraction [10] or electron microscopy [16] can only be performed ex vivo. A common general characterization of the skin barrier function in vivo is the measurement of the transepidermal water loss (TEWL) [17,18,19,20], which is an indirect method and records the amount of unbound water [21] that evaporates out of the skin, but accounts only for the inside-to-outside barrier [6] and includes no depth resolved information. Although TEWL has been applied in several studies determining the effect of topical application on the skin barrier function [22,23,24], it can only be applied on untreated skin, as occlusion effects and water in the formulation can severely influence the measurement results. Closely related is the water binding capability in the SC, which has to consider the total water in the SC that further consists of tightly-, strongly-, weakly-, very weakly-bound water and free water based on the strength of hydrogen bonds to the surrounding molecules [25].

Water in the SC is bound to the polar head groups of the lipids inside ICL lamellas, but is mostly located inside the corneocytes [26,27], where it is efficiently bound to highly hygroscopic natural moisturizing factor (NMF) molecules [28], originating from the breakdown of filaggrin during the cell differentiation process [29] and to keratin [30,31,32]. An increase in NMF content in the corneocytes could potentially increase the amount of bound water in the SC. The lateral lipid organization, which is responsible for SC permeation and the water binding properties of NMF represent the two major factors in providing skin hydration [27,33]. Among other cosmetic substances [34,35,36], the topical application of an NMF containing substance is an important factor for improvement of the skin barrier function.

Recently, several skin barrier related parameters, characterizing the ICL organization [11], water binding properties [25], folding of keratin [32] and NMF content [37,38,39] were determined depth dependently in the SC using in vivo confocal Raman microspectroscopy. Here, it has been shown, that these parameters are inhomogeneous, exposing a maximal lipid ordering in the depth 20–40% of the SC thickness, which correlates with the NMF content and the hydrogen bonding state of water. The SC swelling is also not homogenous and only the intermediate SC depths were found to be responsible for swelling of the SC [32,40], as it is most probably due to the loosing of hydrogen bonds of strongly bound water, which increase the concentration of weakly bound water due to the occlusion [41]. Age-dependent differences have also been reported [42].

Additionally, to healthy untreated human skin, the parameters were determined in skin topically treated with oils, which did not penetrate deep into the SC [43], but acted as an occluding medium, preventing from dehydration [41]. The influence of oils on barrier function-related parameters of the SC were determined in in vivo experiments [44]. Similarly, using CRM, differences of the barrier function-related parameters in barrier disrupted skin in patients with atopic dermatitis [45] and psoriasis [46] were investigated.

In the presented randomized, placebo-controlled, double-blind study, the evaluated parameters were determined after topical application of a moisturizing formulation containing 1% of a proprietary mixture based on the composition of NMF. The measurements were performed 1 h, 4 h, 7 and 14 days after the beginning of the daily treatment, in order to examine short-term and long-term effects of increased water binding capabilities to the skin barrier related parameters.

## 2. Results and Discussion

**Figure 1:**
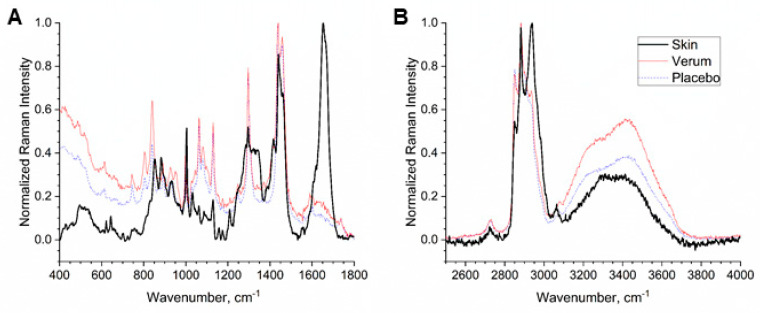
Normalized mean Raman spectra of untreated human stratum corneum (SC) in vivo in 3.4 µm depth (black), the verum (red) and placebo formulations (blue) in the fingerprint (FP, **A**) and high wavenumber (HWN, **B**) regions.

Figure 1 shows the normalized Raman spectra of untreated human SC measured in vivo in 3.4 µm depth (black), the verum (red) and placebo (blue) formulations in the fingerprint (FP, Figure 1A) and high wavenumber (HWN, Figure 1B) regions. Raman spectra of both formulations are visually similar and it is impossible to conclude which is the verum and which the placebo. Since both formulations have a strong contribution in the 2800–3000 cm^−1^ range, which is specific to lipids and keratin in the SC, the calculation for the lipid and total water concentration, which are normalized to the keratin concentration, could be affected in the superficial depths [47].

### 2.1. SC Thickness

The SC thickness can be regarded to be a characteristic measure of the skin barrier function, especially if the amount of lipid bilayers, maintaining the skin barrier, is increased. However, considering the SC thickness alone as a parameter for the skin barrier function is questionable due to a possible formulation-induced swelling effect. An average SC thickness of 22.1 ± 2.1 µm was determined for untreated skin.

**Figure 2:**
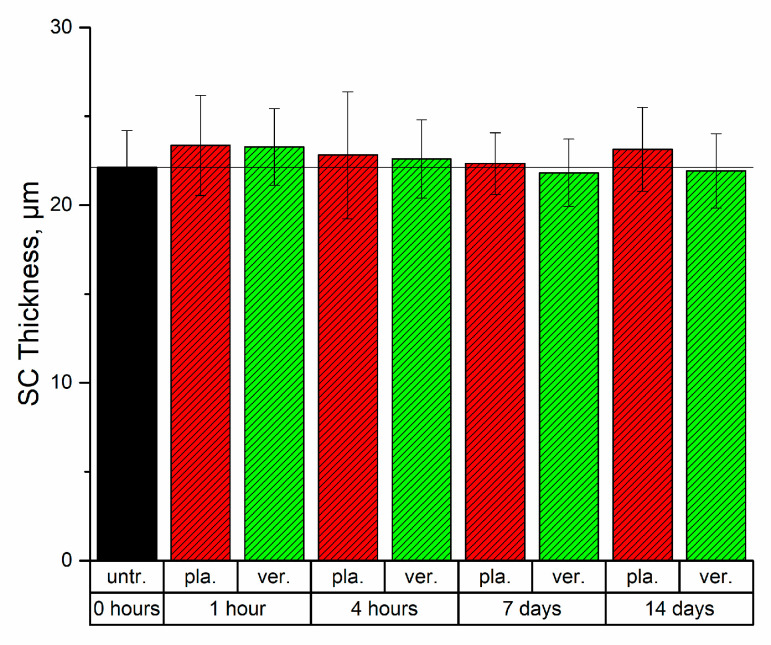
Average stratum corneum (SC) thickness for untreated skin (black) and skin treated with the placebo (red) and verum (green) after 1 h, 4 h, 7 days and 14 days after beginning of the treatment. The differences of all treated SC thicknesses compared to untreated SC were strongly significant (*p* < 0.01), no significant differences were found between placebo and verum. The data show mean ± standard deviation.

As a short-term effect, after 1 h the SC thickness increases by 1.2 µm and 1.3 µm to a total thickness of 23.3 ± 2.2 µm for the verum and to 23.4 ± 2.8 µm for the placebo, respectively. After 4 h the total thickness of the SC was 22.6 ± 2.2 µm for the verum and 22.8 ± 3.6 µm for the placebo. All changes were significant (*p* < 0.01). This increase could be due to an occlusion effect directly after treatment, reducing the evaporation of water and resulting in swelling of the SC [41,48] (Figure 2).

As a long-term effect, the SC thickness decreased to a total thickness of 21.8 ± 1.9 µm after 7 days and 21.9 ± 2.1 µm after 14 days for the verum and the total SC thickness increased to 22.3 ± 1.7 µm after 7 days and to 23.1 ± 2.4 µm after 14 days after application of the placebo (Figure 2). In this case, a direct occlusion effect would be excluded, since the formulations were not applied on the day of the measurements before the experiments. The changes were significant (*p* < 0.01) and could be attributed to the osmotic contribution of the NMF molecules. Twice daily application of the verum formulation containing NMF molecules could allow for the possibility of these molecules to penetrate deeper in the SC and bind water. However, the retention of these molecules on the surface of the skin cannot be excluded and would need further investigations.

### 2.2. Penetration Depth

A penetration of the formulations could only be observed as a short-term effect. For the placebo formulation, a penetration to 40% SC depth after 1 h and 20% SC depth after 4 h was observed. Similarly, for the verum penetration depths of 30% SC depth after 1 h and 10% SC depths after 4 h were observed. A possible explanation for the slightly lower penetration of the verum formulation could be the water binding capabilities of NMF molecules [49]. If the number of NMF-water clusters increase in the lipid lamella, the penetration could possibly be interrupted and hence, be reduced.

The volunteers have not applied any formulations directly before the measurements 7 and 14 days after the beginning of the study, but in these cases, the formulation was applied on the previous evening before the measurements. Here, a penetration depth could not be determined as a long-term effect. This could be explained by the longer time period between the last application and the measurement, which would mean the penetrated formulation alteration due to the contact with SC from one side and desquamation due to epidermal renewal from the other side. The epidermal renewal entails a loss of ≈1 corneocyte layer per day [50] out of the ≈10–15 layers of corneocytes, of which the SC consists [30]. A further, albeit unlikely explanation could be effects of washing and contact to clothes for longer time periods [51].

### 2.3. NMF Content

**Figure 3:**
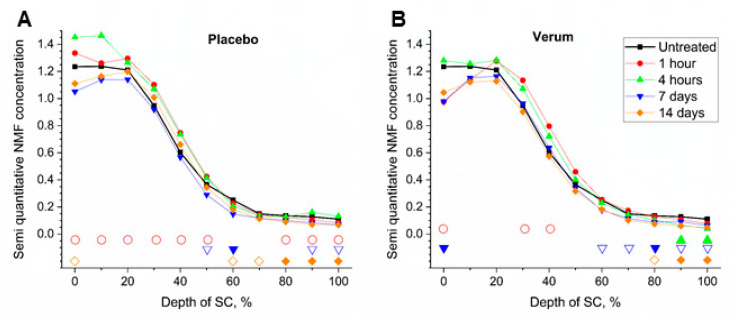
Mean content of natural moisturizing factor (NMF) of all subjects before (untreated, black squares) and 1 h (red circles), 4 h (green upward triangles), 7 days (blue downward triangles) and 14 days (orange diamonds) after beginning of the treatment with the placebo (**A**) and verum (**B**). The signals are normalized to the thickness of the SC (0–100%). Significant differences (*p* < 0.05) are marked below in empty shapes, strongly significant differences (*p* < 0.01) in solid shapes.

Figure 3 shows the NMF concentration profiles in the SC. In untreated SC, a maximum occurred in ≤20% SC depths, while the lowest NMF concentration occurred at the boundary between the SC and the stratum granulosum (SG), which is in agreement with previous results [25,32]. The treatment resulted in significant changes in NMF concentration in different SC depths.

In placebo-treated skin, when compared to the untreated measurement, the NMF content increased as a short-term effect by 8.1% (*p* = 0.035) after 1 h in the upper 20% of the SC thickness (Figure 3). This was not observed in the verum treated skin. To explain this, we hypothesize that the naturally present NMF molecules of the SC were drawn into the water of the formulation causing a redistribution of NMF throughout the SC similar to the dilution of NMF in the occluded SC due to oil treatment [41]. This is based on previous studies [40,52] and taking into consideration that these highly aqueous soluble molecules are present in a lipophilic environment in the SC. This redistribution was quelled within 4 h, leaving a significant increase of NMF at the surface. For the verum, the NMF content at the SC surface decreased by 20.3% (*p* = 0.034) after 1 h and did also not change after 4 h. This could be explained by the fact that the verum formulation already contained l-Serine, l-Argenin, l-Histidine, l-PCA and Calcium lactate, which are components of NMF. This limited the amount of NMF drawn out of the SC and supported this natural moisturizer instead. The significant decrease in NMF in the deepest layers of the SC at 4 h for verum treated skin and not in the case of the placebo could indicate, either a slower rebalancing of the NMF profile is needed in the case of the verum or, albeit unlikely, that components with low concentrations of the verum mixture had reached the living epidermis taking with them some of the NMF. However, this data should be interpreted with caution as the method used reference spectra of l-PCA, ornithine, serine, proline, glycine, histidine and alanine [53], which partly match the components of the verum formulation.

As a long-term effect, since the last application was performed ≈12 h prior to the measurement, the loss of NMF at the skin surface caused by it being drawn out is made even clearer for both, the placebo, as well as the verum formulation. This loss was also manifested as a significant decrease in NMF at 60–100% SC depth for the placebo and at 80–100% SC depth for the verum after 14 days treatment.

Since the verum contains components of NMF, it would be expected, that the detected NMF concentration increased for the verum and not for the placebo as a short-term effect. This contradiction could be explained by the calculation method. In skin tools, reference spectra of pyrrolidone carboxylic acid, ornithine, serine, proline, glycine, histidine and alanine were used [53], while the components in the verum are L-Serin, L-Arginin, L-Histidin, L-PCA and Ca^2+^ L-Lactate. Therefore, a large part of the NMF components in the verum could not be detected, which results in the absence of NMF increase in the superficial SC depths. The statistically significant differences obtained in the bottom layers of the SC could be artifactual.

From the observations in this study, we hypothesize that the calcium containing supplementation of NMF would enable the formation of desmosomes, which would be eventually matured to corneodesmosomes. This results in a SC where corneocytes are bound to each other more tightly, offering a further explanation to the reduction of the SC thickness, which was observed as a long-term effect [54,55] (Figure 2).

### 2.4. Lipids

#### 2.4.1. Total Lipid Concentration

The lipid concentration in the SC, determined by the lipid/keratin ratio was maximal at the skin surface, where it was ≈0.55 and gradually decreased to the bottom of the SC, reaching ≈0.4 for untreated skin, which is in agreement with previous results [11]. An increase of the lipid concentration after treatment, especially as a short-term effect in the upper layers of the SC (0–20% SC depth) was observed (≤36% increase after 1 h, ≤24% after 4 h, ≤9% after 7 and 14 days). Significant differences of untreated to placebo treated skin occurred infrequently at 60% (increase), 90% (decrease) and 100% depth (decrease) after 1 h (*p* = 0.0235, 0.0262 and 0.0412, respectively) and at 50% depth after 14 days (increase, *p* = 0.0428); after 4 h, the increase at 50% was strongly significant (*p* = 0.00346). This observed increase was likely an effect of the formulations, containing lipids, specifically isopropyl palmitate and emollients components like cetearyl alcohol and isononyl isononanoate, which have Raman peaks at 2850 and 2880 cm^−1^, as shown in Figure 1B. The lipid concentration of the directly measured placebo formulation (1.34 ± 0.09) was slightly higher than that of the verum formulation (1.16 ± 0.08), but both formulations had considerably higher values than the untreated skin on the SC surface (0.56 ± 0.03). Thus, this increase in the superficial layers after formulation application appears reasonable.

#### 2.4.2. Lateral ICL Organization (S_lat_)

**Figure 4:**
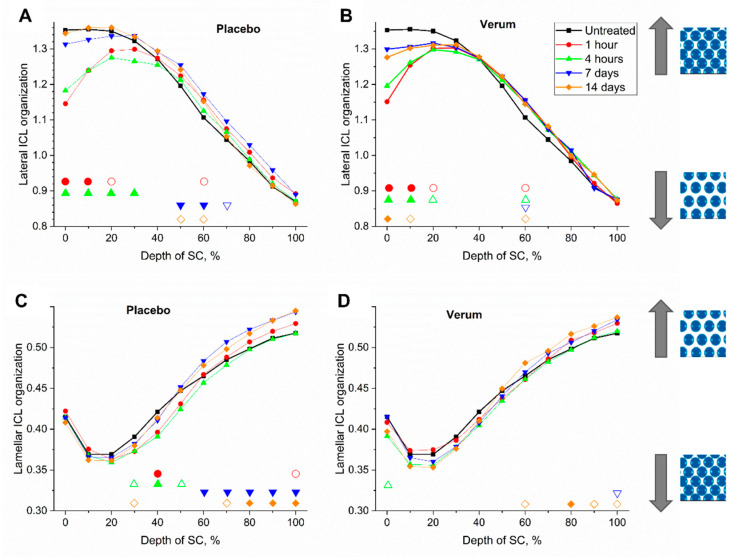
Mean lateral (**A**,**B**) and lamellar (**C**,**D**) organization of intercellular lipids (ICL) of all subjects before (untreated, black squares) and 1 h (red circles), 4 h (green upward triangles), 7 days (blue downward triangles) and 14 days (orange diamonds) after beginning of the treatment with the placebo (**A**,**C**) and verum (**B**,**D**). The signals are normalized to the thickness of the SC (0–100%). Significant differences (*p* < 0.05) are marked below in empty shapes, strongly significant differences (*p* < 0.01) in solid shapes. 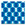
*/*
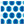
—more or less ordered lateral structure of ICL in the SC.

A short-term *S_lat_* decrease after 1 and 4 h could clearly be observed in SC depths ≤20% (Figure 4A,B). After application of the placebo, this effect could be observed deeper (40% SC depth as opposed to 30% SC depth after application of the verum), which is in agreement with the higher penetration ability of the placebo compared to the verum after 1 h, as mentioned above. The *S_lat_* decrease could be due to the disruption of the lipid organization induced by the formulations or interference from the palmitate in the formulation. Measurements directly on the formulation exposed an *S_lat_* of 1.0 ± 0.2 for the placebo and 0.9 ± 0.1 for the verum. The finding that the superficial *S_lat_* values of the treated skin 1 and 4 h after application were between those of the untreated skin surface (1.35 ± 0.09) and those of the formulations, supports the assumption that the superficial values are due to the lipids of the formulations and not of the formulation-induced ICL changes of the SC. After long-term application (Figure 4A,B), it would appear that the verum treatment limited the lateral disorganization of the ICL to the upper 20% SC depth. Since the trends can be overlaid for all of the time points measured in 50–80% SC depths (increase by up to 5%) with the verum treatment indicates that the orientation in a higher ordered state is a virtue of physical properties of the components of the verum. A significance in the higher lateral organization at 60% SC depth was observed for all time points for the verum, whereas it was less obvious for the placebo. Thus, the improvement in lateral organization cannot be attributed to the verum alone.

#### 2.4.3. Lamellar ICL Organization (S_skeleton_)

An *S_skeleton_* of 0.34 ± 0.06 was determined for the placebo and 0.37 ± 0.05 for the verum, when measuring the formulations directly, which is comparable to the *S_skeleton_* of superficial SC depths in vivo. In Figure 4C,D, for both treatments, as well as in untreated skin, *S_skeleton_* increased from 10–20% SC depth towards the SG that indicates a decrease of the lamellar organization of intracellular lipids. No change in lamellar organization was observed 1 h after the treatment. After 4 h, the placebo treated skin showed significant changes towards a higher ordered state at 30–50% SC depth, which was not seen for the verum. However, after 7 and 14 days of treatment, the placebo treated skin showed transformation of ICL’s lamellar organization towards a *gauche*-conformation at depth of 60–100% and 70–100% SC depth, respectively. This was seen only at the deepest point of measurement for the verum treated skin after 7 days and was limited to deepest 60 and 80–100% SC depth after 14 days.

The shift to a more disorganized state with the placebo treatment could be explained by occlusion-induced swelling and related disruption of lipid organization of the SC depths, as shown by the increased SC thickness of the placebo as a long term effect [41]. A further possibility could be that the factors of the formulation causing lateral disorganization after the first four hours of application caused lamellar disorganization after 12 h of application, since these were the conditions under which the measurements after 7 and 14 days were made. Since serine was present in the verum formulation, disorganization of the lamellar arranges in the depths of the SC could be attributed to an enhanced synthesis and release of ceramides form lamellar bodies of keratinocytes of the SG. This is because serine is a substrate of serine palmitoyltransferase, which is the rate controlling enzyme in the biosynthesis of ceramides [2,56,57,58].

This study determined that the lateral organization of ICL moved from a more disorganized state to a more organized state with increasing SC depth, whereas a partly reverse trend was observed for the lamellar organization of ICL which moved from a more organized state to a more disorganized state in the bottom of the SC. A possible explanation for greater disorganization of lamellar organization at the bottom of the SC could be because of the release of lipids that form the ICL milieu from the lamellar bodies of keratinocytes of the SG. Thus, in the verum—which contains serine—this organization was further disrupted since serine is the substrate of the rate determining ceramide synthesis enzyme, serine palmitoyltransferase. The verum also limited disruption of lateral organization caused by the base formulation to the upper 20% layers of the SC.

### 2.5. Water

#### 2.5.1. Total Water Concentration

The total water concentration was comparable to previous results [32,59]. 1 and 4 h after application of both formulations, only a minor decrease in the upper SC layers, which were not statistically significant was observed. This is an artifact, which could be explained by the presence of the Raman peak in the “keratin range” (2935 + 2980 cm^−1^) of the formulations (see Figure 1B), originating from lipids and proteins, which were used for Raman intensity normalization. The total water/keratin ratio of the placebo is 3.5 ± 1.5, which was slightly higher than that of the verum, where it was 2.5 ± 1.0. Nevertheless, 1 and 4 h after application, it was expected that water contained in the formulation would be mostly evaporated, while lipids and proteins would be present in the superficial layers of the SC, thus substantially lowering the total water/keratin ratio of the formulation itself and of the treated skin, as recently reported [47]. It has also been found that hydrophilic moisturizers can reduce the water content in the SC. Corneocytes in the swelling SC region (≈30–70% SC depth) are most sensitive to the moisturizers [40].

#### 2.5.2. Tightly Bound Water

**Figure 5:**
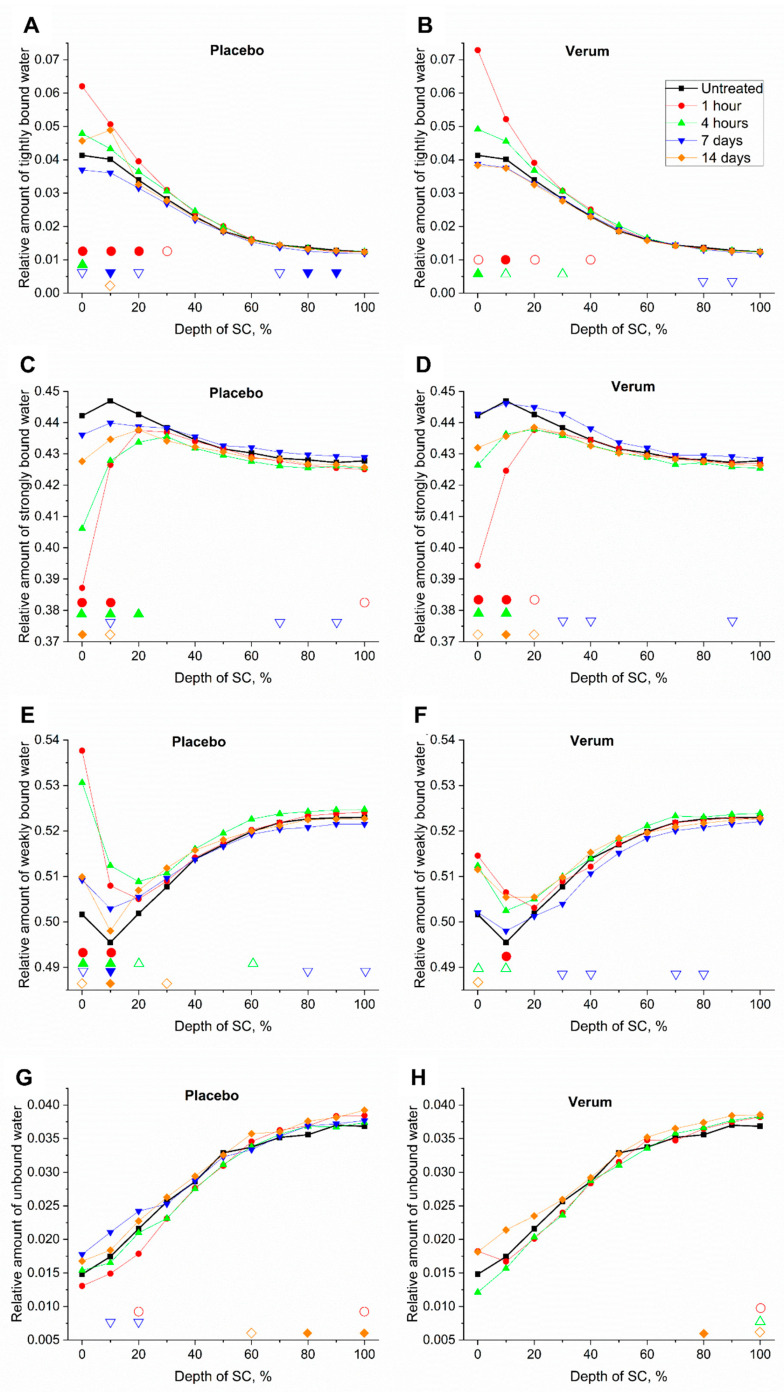
Relative mean amount of tightly bound—(**A**,**B**), strongly bound—(**C**,**D**), weakly bound—(**E**,**F**) and unbound water (**G**,**H**) of all subjects before (untreated, black squares) and 1 h (red circles), 4 h (green upward triangles), 7 days (blue downward triangles) and 14 days (orange diamonds) after beginning of the treatment with the placebo (**A**,**C**,**E**,**G**) and verum (**B**,**D**,**F**,**H**). The signals are normalized to the thickness of the SC (0–100%). Significant differences (*p* < 0.05) are marked below in empty shapes, strongly significant differences (*p* < 0.01) in solid shapes.

Figure 5A,B shows a superficial increase of tightly bound water. As a long-term effect (14 days) the increase after application of the placebo was stronger than after application of the verum. The relative amount of tightly bound water of the directly measured formulations was 0.004 ± 0.002 for the verum and 0.002 ± 0.001 for the placebo, which can slightly influence the results. A slight increase of tightly bound water 1 h and 4 h post-application of both formulations in the superficial SC layers can be explained by the evaporation of more loosely bound water from the formulations with no changes of tightly bound water in the remaining formulations.

#### 2.5.3. Strongly Bound Water

Figure 5C,D shows a superficial decrease of strongly bound water, especially as a short-term effect. This could be explained by a formulation-induced occlusion effect, preventing the loosely bound water from evaporating and therefore accumulating in the upper SC layers. Due to the increase of loosely bound water in the superficial SC layers, the percentage of strongly bound water decreased, as an effect of “dilution”, for both formulations. Another explanation could be an effect of water content in the applied formulations, entailing an increase of total water, while the amount of strongly bound water remains constant. However, the relative amount of strongly bound water of the placebo formulation itself (0.43 ± 0.01) was slightly higher than that of the verum formulation (0.41 ± 0.01) and can potentially influence the superficial values. This effect is probably less pronounced due to water evaporation from the formulations post application on the skin.

In the depths 20–40% of the SC thickness, where the maximal skin barrier occurs [11,25], the concentration of strongly bound water increased by ≤1% after 7 days of verum treatment, while it varied only slightly after placebo application. After 14 days, a decrease of ≤1% was observed, which was always slightly smaller for the verum.

#### 2.5.4. Weakly Bound Water

Figure 5E,F shows a superficial increase of weakly bound water. This could be explained by an occlusion effect, preventing the loosely bound water from evaporating, therefore entailing accumulation in the upper SC layers. The amount of weakly bound water was higher in the upper SC layers after application of the placebo than after application of the verum as a short-term effect. The superficial increase was unlikely due to the relative amount of weakly bound water of the placebo (0.53 ± 0.01) and the verum formulations (0.54 ± 0.01), since they showed an opposing tendency and formulation-related water was, probably, evaporated quickly post application. The effects of weakly bound water always showed an opposing tendency to strongly bound water, which showed that the topical application mostly influenced water with these two hydrogen bonding types. Further, weakly and strongly bound water make up >90% of the total water.

In the depths of the maximal skin barrier function (20–40%) [11,25], the concentration of weakly bound water decreased by ≤0.75% after 7 days of verum treatment, while it varied only slightly after placebo application. After 14 days, an increase of ≤1.2% was observed, which is always smaller for the verum.

#### 2.5.5. Unbound Water

Figure 5G,H shows only slight fluctuations in the unbound water content after treatment. The relative amount of unbound water of the placebo (0.039 ± 0.003) and the verum (0.041 ± 0.004) had only a small influence on that of the SC at its surface, as this water was evaporated immediately after an application on the skin. The occurrence of single significant differences should be considered carefully.

### 2.6. Hydrogen Bonding State of Water Molecules

**Figure 6:**
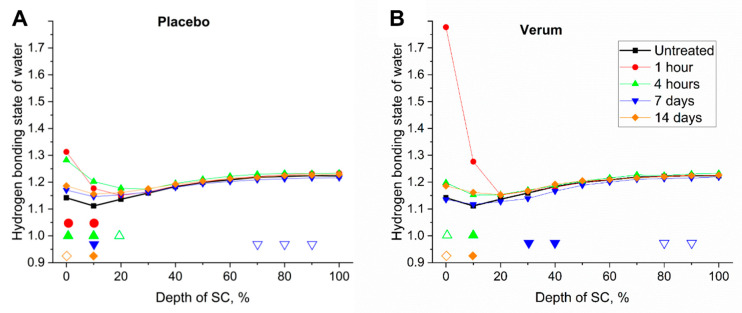
Mean hydrogen bonding state of water (weakly/strongly bound water ratio) of all subjects before (untreated, black squares) and 1 h (red circles), 4 h (green upward triangles), 7 days (blue downward triangles) and 14 days (orange diamonds) after the beginning of the treatment with the placebo (**A**) and verum (**B**). The signals are normalized to the thickness of the SC (0–100%). Significant differences (*p* < 0.05) are marked below in empty shapes, strongly significant differences (*p* < 0.01) in solid shapes. A higher hydrogen bonding state determines a higher amount of more loosely bound water, where the water is most loosely bound with surrounding molecules.

In Figure 6, a superficial increase (<20% of SC depth) can be observed, especially 1 h after application of the verum. This could be explained by an occlusion effect of the formulations, preventing more loosely bound water from evaporating. This effect was less pronounced for the application of the placebo. Since NMF is effective in binding water, the amount of weakly bound water could be reduced. This is, however, not the case for the verum. Since the hydrogen bonding state of the placebo is 1.23 ± 0.04 and that of the verum is 1.30 ± 0.06, the strong increase at the surface was unlikely to be an inherent effect of the formulations, as this could only slightly increase the hydrogen bonding state of water of the SC to the inherent level of the formulations. Moreover, water evaporation from the formulations should decrease the hydrogen bonding state of water, originating from the formulations.

In the depths of maximal skin barrier function (20–40% of SC thickness), the placebo showed an increase of the hydrogen bonding state of water, while the verum showed a decrease after 7 days, which increased more after 14 days.

Our results confirm the recently published observation that occlusion induces the transformation of water binding from strongly bound towards weakly bound in the superficial SC region [41].

## 3. Materials and Methods

### 3.1. Study Protocol

Twelve volunteers (aged 25–58, mean 38.8), consisting of six female (aged 27–58, mean 41.2) and six male (aged 25–53, mean 35.8) of skin type II–III according to Fitzpatrick classification [60] without any reported skin diseases participated in this randomized, placebo-controlled, double-blind study. The volunteers marked a 4 × 4 cm^2^ area on their inner forearms and applied a verum and placebo formulation provided by Merck KGaA (Darmstadt, Germany) twice a day (usually, in the morning and evening) for 14 days in the marked areas. Six volunteers applied the verum on the left and the placebo on the right volar forearm and the remaining six volunteers vice versa. During the study, both formulations were arbitrarily labeled as “A” and “B”, guaranteeing double-blind experiments for the volunteers and examiners. The placebo formulation was an oil in water base formulation. In addition to the base formulation components, the verum formulation contained a 1% Mixture (RonaCare^®^ Epintegrity), comprising l-Serine, l-Argenin, l-Histidine, l-PCA and calcium lactate. The effect of the base formulation without these NMF components was investigated as a control (placebo). The components of the formulations are listed in the Supplementary Materials in Table S1.

For the first application, the formulations were weighted and 32 mg was homogeneously applied to the marked skin areas, resulting in 2 mg/cm^2^. For the further applications, the volunteers were instructed to apply a pea-sized amount of formulation without weighing, which was enough to cover the marked areas. On average, the volunteers applied 5 ± 3 g of formulation on each area within 14 days. The volunteers were instructed not to use any other skin care products on the forearms two weeks before and during the study.

### 3.2. Confocal Raman Microspectroscopy (CRM)

In vivo confocal Raman microspectroscopy (CRM) was performed before the first application (untreated), 1 h, 4 h (short-term) and 7 days and 14 days (long-term) after the first application on both volar forearms in a standardized laboratory environment at 21 °C. The measurements after 7 and 14 days were performed before the first formulation application of the day. The CRM depth profiles were acquired using the Model 3510 skin composition analyzer (RiverD International B.V., Rotterdam, the Netherlands) [61,62] in the FP (20 mW excitation at 785 nm, detection at 400–2000 cm^−1^) and HWN (17 mW excitation at 671 nm, detection at 2500–4000 cm^−1^) regions, consisting of Raman spectra from above the skin surface down to a depth of 40 µm in the skin in 2 µm increments, which is distinctly below the SC. The CRM has a depth resolution of ≤5 µm and a lateral resolution of 1 µm. On each forearm, 7–8 depth profiles have been recorded.

### 3.3. Data Analysis

All data analysis was performed in MATLAB R2016 (MathWorks, Nattick, MA, USA) and Origin 9.1 (OriginLab Corporation, Northampton, MA, USA). For pre-processing, cosmic spikes were detected by analyzing the intensity difference between neighbored sampling points and were replaced by the median of the three neighbored ones. The fluorescence background was removed by iteratively subtracting the minimum between the spectrum and a fitted fifth order polynomial to the spectra [63] in the FP region and subtracting a combination of a weighted second order polynomial (weight = 1: 2776–2810 cm^−1^ and 3700–3900 cm^−1^; weight = 0.1: 3034–3042 cm^−1^) and a linear function (3034–3900 cm^−1^) in the HWN region [25]. The low variability components, which were assumed to be related to noise, were removed by reconstruction of the spectral profiles using the first four components of principal component analysis [64].

The skin surface of each profile was determined based on half of the maximal keratin profile in the FP region (1650 cm^−1^ Amide I Raman peak) [37,65,66] since no bands of the formulations were superimposing with the skin spectra in this region. Among different methods to determine the SC thickness [67,68,69] using CRM, in this study, it was calculated by determining the border between the SC and the stratum granulosum to be at the position, where the profile of the water related band in the HWN region (3350–3550 cm^−1^) reached a gradient value of 0.5 [70]. All depths were subsequently normalized to the SC thickness and the further calculated parameters were interpolated to 10% increments of the SC thickness. In order to compensate the depth dependent signal attenuation due to scattering and absorption of the skin, the analyzed individual Raman bands were normalized to the keratin signal.

A decomposition procedure was performed in the 2650–3800 cm^−1^ broad band wavenumber range using 10 Gaussian functions to analyze the contribution of superimposed Raman bands of keratin, lipids and water characterized with different strength of hydrogen bonds (Table 1).

For clarity, all diagrams are shown without error bars. Significant differences (*p* < 0.05) between treated and untreated skin are marked at the bottom of the diagrams in empty shapes and strongly significant differences (*p* < 0.01) are marked in filled shapes for different SC depths. The effects of multiple testing, resulting in increased Type-I error, were neglected.

#### 3.3.1. Penetration Depth and Content of Natural Moisturizing Factor (NMF)

The penetration depth of the applied formulations and the NMF content were determined using the Skin Tools 2.0 software (RiverD International B.V., Rotterdam, the Netherlands), which is based on linear regression of reference spectra of various SC components (keratin, lactic acid at pH4, urea, trans-urocanic acid at pH4 and pH8, ceramide type III, cholesterol and water) and spectra related to the optical components of the CRM [53,61], as well as previously acquired reference spectra of the applied formulations shown in Figure 1.

The NMF concentration was determined as a combination of the pyrrolidone carboxylic acid, ornithine, serine, proline, glycine, histidine at pH4 and pH7 and alanine concentrations using a least-squares fitting procedure incorporated in the Skin Tools 2.0 software [61]. Thereby, the semi-quantitative NMF-concentration determines the relative amount of the NMF-related signal contribution to the total Raman signal for every measured depth in the SC.

#### 3.3.2. Intercellular Lipids (ICL)

The total lipid concentration was determined by the sum of the lipid-related 2875–2885 cm^−1^ and 2845–2855 cm^−1^ sub-band AUCs (area under the curve), which were modified in order to exclude the influence of keratin on the lipid related peaks and to compensate the depth-dependent signal attenuation due to light scattering and absorption in the skin, using a previously published algorithm [11].

Similarly, the lateral ICL organization [9] (*S_lat_*) is determined by the ratio of these sub-band AUCs (*S_lat_* = *AUC_2880_*/*AUC_2850_*). A higher *S_lat_* denotes an increase of orthorhombic organization phase (denser ICL lateral organization) and thereby a higher skin barrier function, which is known to be non-homogeneous in the SC in vivo with a prominent maximum at 20–40% SC depth [11,59].

The lamellar ICL organization (*S_skeleton_*) is determined by the *S_skeleton_* = *AUC_1076–1096_*/(*AUC_1058–1070_* + *AUC_1128–1138_*) ratio of the Raman band AUCs in the FP region [9,11,71]. The bands around 1060 and 1130 cm^−1^ are sensitive to a *trans*-conformation, while the 1080 cm^−1^ band is sensitive to a *gauche*-conformation of the lipid tail groups. Therefore, a higher *S_skeleton_* determines an increase of the amount of lipid chains in the *gauche*-conformation and thereby indirectly a less ordered ICL organization in the lamellas and hence, a lower skin barrier function. Vice versa, a lower *S_skeleton_* determines an increase of lipid chains in the *trans*-conformation and a more dense and compact packing of the lipids [21] and thus, indirectly, a higher skin barrier function.

#### 3.3.3. Water Concentration and Hydrogen Bonding State of Water

The total water concentration is determined by the ratio of the AUCs of the water-related band (3350–3550 cm^−1^) to the keratin-related band (2935–2980 cm^−1^), which is calculated to compensate the depth-dependent signal attenuation due to light scattering and absorption in the skin.

The relative amount of the water with different strengths of hydrogen bonds in the SC, listed in Table 1, was calculated by the ratio of the intensity of the decomposed band to the sum of all four water-related sub bands [25,72,73]. Tightly bound water was associated to the 3015 cm^−1^, strongly bound water to the 3225 cm^−1^, weakly bound water to the 3451 cm^−1^ and unbound water to the 3633 cm^−1^ sub band. In normal untreated skin, tightly bound and unbound water each constitute <5%, while strongly and weakly bound water each constitute >45% of the total water in the SC [25].

The hydrogen bonding state of water is determined by the ratio of the decomposed band intensities sensitive to weakly (3451 cm^−1^) and strongly (3225 cm^−1^) bound water, respectively. Therefore, a higher value of a hydrogen bonding state in the SC determines a higher amount of more loosely bound water (SC depths, where the water is most loosely bound with surrounding molecules) and a lower value determines more strongly bound water (SC depths, where the water is most strongly bound with surrounding molecules) [25,42].

#### 3.3.4. Statistical Analysis

The calculated Raman parameters for both formulations at every visit (1 h, 4 h, 7 days and 14 days) were compared to those of untreated skin at the beginning of the study for every SC depth using paired *t*-tests. *p*-values < 0.05 were considered significant and *p*-values < 0.01 were considered strongly significant.

## 4. Conclusions

The effects of a topically applied formulation on the skin can be monitored using CRM. The applied formulations disrupted the equilibrium of water, NMF and lipids in the SC. The adverse effects of the formulation could be mitigated by incorporating it into a simplified supplementation of NMF molecules. This supplement was unexpectedly also able to prevent ICL disorganization in selected depths. Although, further research is required to elucidate the interplay of these factors in the SC, this study already reveals first insights into the contribution of NMF to the equilibrium and barrier function of the skin.

As a long-term effect, the amount of strongly bound water increased at 30–40% SC depth (*p* < 0.05) for the verum. A strong tendency was also observed at 20% SC depth. The amount of weakly bound water decreased at 30–40% SC depth (*p* < 0.05). A strong tendency was also observed at 20% SC depth. The hydrogen bonding state of water decreased at 30–40% SC depth (*p* < 0.01) after verum application. A strong tendency was also observed at 20% SC depth. It can be assumed, that water was more efficiently bound after verum than after placebo application in the depths that expose the maximal skin barrier function (depths 20–40% of the SC thickness).

CRM proved to be a versatile tool to investigate parameters of the SC in vivo in a reliable, non-invasive manner. It offers insight into changes in structural characteristics in addition to the dynamics of physico-chemical processes. Thus, this method has the advantage over other noninvasive methods, like measurement of the TEWL and corneometry by offering depth-dependent component-specific information. Being in vivo and non-invasive CRM has an advantage over methods like (cryo-) electron diffraction, differential scanning calorimetry, X-ray diffraction or electron microscopy, which require the excision of tissue biopsy samples. In recently published literature, CRM has been used to depth-dependently differentiate the binding states of water, the organization of the ICL and the interplay between NMF and keratin for water binding in the SC.

## Figures and Tables

**Table 1:** Associations of the SC’s Raman bands in the high wavenumber (HWN) region calculated by decomposition using Gaussian functions.

Center Position [cm^−1^]	Optimi-Zation Range [cm^−1^]	FWHM [cm^−1^]	Optimi-Zation Range [cm^−1^]	Band Association
2850	2845–2855	20	16–24	Symmetric CH vibration: lipids
2880	2875–2885	37	32–42	Asymmetric CH vibration: mostly lipids, also keratin
2935	2930–2940	52	42–65	Symmetric CH_3_ stretch vibrations: mostly keratin, also lipids
2980	2975–2985	35	30–40	Asymmetric CH_3_ stretch vibrations: keratin
3015	3005–3020	30	20–50	Tightly bound water
3065	3020–3090	40	20–100	CH stretch vibrations: olefin
3225	3190–3242	220	120–265	Strongly bound water
3330	3310–3350	40	10–100	NH vibration: keratin
3451	3450–3515	200	150–240	Weakly bound water
3633	3602–3645	80	50–120	Unbound water

## Data Availability

Data is available from the authors upon reasonable request.

## Supplementary Materials

The following are available online, Table S1: Components of the applied formulations.

## Sample Availability

Not available.
