# Supplementary material for: Retaining Skin Barrier Function Properties of the Stratum Corneum with Components of the Natural Moisturizing Factor—A Randomized, Placebo-Controlled Double-Blind In Vivo Study
Source: Molecules. 2021 Mar 16;26(6):1649. doi: 10.3390/molecules26061649 (PMC8000920; doi:10.3390/molecules26061649)
Supplement: Supplementary file 1 [file molecules-26-01649-s001.pdf]

molecules

MDPI

Supplementary Material to

Retaining skin barrier function properties of the stratum corneum with components of the natural moisturizing factor – a randomized, placebo-controlled double-blind *in vivo* study

Johannes Schleusener [1], Andrew Salazar [2], Jörg von Hagen [2], Jürgen Lademann [1] and Maxim E. Darvin [1,*]

[1] Charité – Universitätsmedizin Berlin, Corporate Member of Freie Universität Berlin and Humboldt-Universität zu Berlin, Center of Experimental and Applied Cutaneous Physiology, Department of Dermatology, Venerology and Allergology, Charitéplatz 1, 10117, Berlin, Germany; johannes.schleusener@charite.de (J.S.); juergen.lademann@charite.de (J.L.); maxim.darvin@charite.de (M.D.)
[2] Merck KGaA, Frankfurterstr. 250, 64293 Darmstadt; joerg.von.hagen@merckgroup.com (J.v.H.); andrew.salazar@merckgroup.com (A.S.)
* Correspondence: maxim.darvin@charite.de

Table S1. Components of the applied formulations.

Ingredients	INCI (EU)	[%]
Montanov 68	CETEARYL ALCOHOL, CETAEARYL GLUCOSIDE	*4.00*
Span 60	SORBITAN STEARATE	*1.50*
Lanette® O	CETEARYL ALCOHOL	*1.00*
Isopropyl Palmitate	ISOPROPYLPALMITATE	*3.00*
Cetiol® CC	DICAPRYLYL CARBONATE	*3.00*
Lanol 99	ISONONYL ISONONANOATE	*3.00*
Water, demineralized	AQUA	*59.40*
1,2-Propanediol	PROPYLENE GLYCOL	*3.00*
Rhodicare XC	XANTHAN GUM	*0.30*
Citric acid anhydrous	CITRIC ACID	*0.10*
tri-Sodium Citrate dihydrate	SODIUM CITRATE	*4.70*
Euxyl® PE 9010	PHENOXYETHANOL, ETHYLHEXYLGLYCERIN	*1.00*
Water, demineralized	AQUA	*15.00*
proprietary mixture based on the composition of NMF (Verum) OR Water, demineralized (Placebo)	SERINE, CALCIUM LACTATE, PCA, ARGININE, HISTIDINE (Verum) OR AQUA (Placebo)	*1.00*
		100.00
